# Cost of maternal health services in selected primary care centres in Ghana: a step down allocation approach

**DOI:** 10.1186/1472-6963-13-287

**Published:** 2013-07-26

**Authors:** Maxwell Ayindenaba Dalaba, Patricia Akweongo, Germain Savadogo, Happiness Saronga, John Williams, Rainer Sauerborn, Hengjin Dong, Svetla Loukanova

**Affiliations:** 1 Institute of Public Health, University of Heidelberg, Heidelberg, Germany; 2 Navrongo Health Research Centre, Navrongo, Ghana; 3 University of Ghana, School of Public Health, Accra, Ghana; 4 Behavioural Sciences Department, Muhimbili University of Health and Allied Sciences, School of Public Health and Social Sciences, Dar es Salaam, Tanzania; 5 Centre for Health Policy Studies, School of Public Health, Zhejiang University School of Medicine, Hangzhou, China

**Keywords:** Cost, Step-down allocation approach, Antenatal care, Delivery, Maternal health service, Ghana

## Abstract

**Background:**

There is a paucity of knowledge on the cost of health care services in Ghana. This poses a challenge in the economic evaluation of programmes and inhibits policy makers in making decisions about allocation of resources to improve health care. This study analysed the overall cost of providing health services in selected primary health centres and how much of the cost is attributed to the provision of antenatal and delivery services.

**Methods:**

The study has a cross-sectional design and quantitative data was collected between July and December 2010. Twelve government run primary health centres in the Kassena-Nankana and Builsa districts of Ghana were randomly selected for the study. All health-care related costs for the year 2010 were collected from a public service provider’s perspective. The step-down allocation approach recommended by World Health Organization was used for the analysis.

**Results:**

The average annual cost of operating a health centre was $136,014 US. The mean costs attributable to ANC and delivery services were $23,063 US and $11,543 US respectively. Personnel accounted for the largest proportion of cost (45%). Overall, ANC (17%) and delivery (8%) were responsible for less than a quarter of the total cost of operating the health centres. By disaggregating the costs, the average recurrent cost was estimated at $127,475 US, representing 93.7% of the total cost. Even though maternal health services are free, utilization of these services at the health centres were low, particularly for delivery (49%), leading to high unit costs. The mean unit costs were $18 US for an ANC visit and $63 US for spontaneous delivery.

**Conclusion:**

The high unit costs reflect underutilization of the existing capacities of health centres and indicate the need to encourage patients to use health centres .The study provides useful information that could be used for cost effectiveness analyses of maternal and neonatal care interventions, as well as for policy makers to make appropriate decisions regarding the allocation and sustainability of health care resources.

## Background

Reducing maternal and under-five mortality through the use of cost-effective strategies continues to be a challenge, particularly in developing countries. The worldwide maternal mortality ratio (MMR), or the number of women who die during pregnancy and childbirth per 100,000 live births, declined from 299 in 1990 to 202 in 2011, representing a 1.9% average annual rate of decline. Globally, under-five mortality also declined over the past years reaching 7.2 million in 2011 [1].

In Ghana, the MMR declined from 394 deaths per 100,000 live births in 1990 to 328 deaths per 100,000 live births in 2011, a 0.9% average annual rate of decline. Also under 5 deaths in the country was estimated at 47,600 deaths in 2011 [1]. In the Kassena-Nankana and the Builsa districts, however, the MMR was high at 367 and 259 maternal deaths per 100,000 live births in 2010 respectively [2,3].

Given the limited health care resources in Ghana, coupled with the wide range of maternal and neonatal care (MNC) services provided free of charge for all women, efficient use of these resources is essential. Despite the need to maximize resources, few cost studies have been conducted in Ghana, and as a result there is little information on the costs of antenatal care (ANC) and delivery services.

This study aims to fill this crucial gap by examining the costs associated with running a health care centre as well as the specific costs associated with ANC and delivery services. This knowledge can better enable health centre managers and policy makers to budget and allocate the appropriate resources that will ensure higher quality of health care services towards reducing maternal and under-five mortality.

## Methods

### Study area

This study was carried out in the Kassena-Nankana and Builsa Districts located in the northern part of Ghana. The Kassena-Nankana District (KND) occupies an area of about 1,675 square kilometres with a population of approximately 150,000 [4]. The district has a hospital, seven health centres, one private clinic and twenty seven Community-based Health Planning and Services (CHPS) compounds with resident Community Health Officers (CHOs) offering doorstep services [5].

The Builsa district lies southwest of the KND and covers an area of 2,220 square kilometres with a population of approximately 95,800. It is served by a district hospital, eight health centres and thirteen CHPS compounds [2].

### Study design

The study has a cross-sectional design and quantitative data was collected from the health provider perspective. Patient costs were not collected and hence not included in the analysis. The study is part of the Quality of Maternal and Prenatal Care (QUALMAT) project, which aims to improve the quality of maternal and prenatal care in Ghana by testing two interventions-a computer-assisted clinical decision support system and performance-based incentives. Details of the QUALMAT project are presented elsewhere [6].

The districts and health centres included in this study were selected by the QUALMAT project based on the criteria that they were typical for the country, and were comparable according to national norms in terms of medical infrastructure, equipment and staffing.

### Selected health care centres

Twelve government run primary health care centres were included in the study (Table 1). In Ghana, health service delivery is organized in five levels: tertiary, regional, district, sub-district (health centres and clinics), and community level. The health care centres included in this study fall under the sub-district level and all met the minimum requirement of having at least one nurse, one midwife, and one medical assistant. The health centres provide outpatient services and normal deliveries.

**Table 1:** Background characteristics of health centres

**Health care centres**	**Year of start of operation**	**Population covered**	**Number of staff**
Paga	1986	13,588	10
Chiana	1966	14,361	18
Kassena-Nankana East (KNE)	2002	15,940	10
Sirigu	1984	8,519	10
Navrongo Health Centre (NHC)	2010	16,003	17
Kolgo	1999	14,361	7
Chuchulga	1998	11,370	10
Kanjaga	2000	11,365	13
Siniensi	1969	7,541	19
Doninga	2003	2,083	4
Wiaga	1950	20,444	22
Fumbisi	2005	15,197	21
**Total**		**150,772**	**161**
**Average**		**12,564**	**13**
**Median**		**13,975**	**10**

Though all the health centres included in this study have the minimum staff, the staff strength at each health centre differs. For instance, whereas the Wiaga Health Centre had a staff strength of 22, that of Doninga Health Centre was only 4 (Table 1). In addition, the year of construction of the health centres and the start of operations differed between the health centres. Whereas some of the health centres started operating in the 1950’s (Wiaga), others were only opened after 2000 (Doninga, Kologo, Fumbisi and Kassena-Nankana East).

Though all the study health centres had basic infrastructure such as utilities (electricity, water), equipment (sphygmomanometer, thermometer, stethoscope, weighing scales etc.), medical supplies (urine testing kits, cannula, giving sets, syringes and needles, cotton, gauze, etc.), vehicles (motorbikes), drugs (amoxicillin, gentamicin, SP, iron, etc.), the out-of-stock items varied between the health care centres.

The catchment areas of the health centres also varied. Doninga health centre covered the smallest population of about 2,083 residents, and Wiaga covered 20,444 residents (Table 1). Though Wiaga is a health centre, it serves as a referral point and sometimes provides inpatient care. The health centre is believed to provide quality care due to it being a missionary facility, run by the Catholic Church. For this reason, a good proportion of patients visiting the Wiaga health centre are not within its catchment area.

### Data collection

Data was collected between July and December 2010. All health care related costs collected in the data covered a one- year period (2010). A standard structured health facility questionnaire was used to collect data and included nine sections: building, equipment, vehicle, human resources/personnel, existing incentives, supplies, operations and maintenance costs (Table 2).

**Table 2:** Cost categories

**Category**	**Description**
**A. Recurrent cost**	
**Personnel cost**	It includes gross income of staff (including volunteer workers), complements of earnings (i.e. housing allowances, overtime, car allowances), human resource costs( cost of training for staff for at least 1 year during the last 3 years) and cost of performance -based incentives (both monetary and value of non-monetary).
**Administrative cost**	It includes electricity, water, telephone bills, cleaning products, repairs, post office, printing, photocopying, and stationary. In addition, cost of spare parts purchased, servicing of the means of transport, fuels and lubricants consumed were also included.
**Pharmacy costs**	These costs included the costs of drugs and vaccines consumed within the period.
**Laboratory costs**	This group included all laboratory supplies used in the period.
**Medical supplies cost**	This group included all medical consumables used in the period.
**B. Capital cost**	
**Cost of building**	It refers to all the rooms in the health care centre. The total area for the various rooms measured was multiplied by the cost per meter square to give the total replacement value. The cost per meter squared was obtained from the Estate officer at the district assembly ($14 US per meter squared).
**Vehicle costs**	These included all vehicle costs (motorcycles, four-wheel vehicles, three wheel vehicles, ambulance and bicycles).
**Equipment cost**	It includes cost of general equipment and equipment in the various rooms (waiting room, consulting room etc.) such as benches, tables, chairs etc.

The data were collected through interviews with health centre staff, document review, physical measuring of rooms and counts of equipment. The prices of items were obtained through store -keeper invoices or from the regional medical stores. In order to calculate staff salaries, the average salaries per staff type were graded as indicated by the Ministry of Health [7]. We determined the percentage work time or workload by staff for each department via interviews. Utilization data, such as number of ANC visits and the number of deliveries at the various health centres, were also collected via document reviews.

Data was entered in Epi info version 3.5.1 and analyzed using Microsoft Office Excel 2007. Ethical clearance was obtained from the ethics committee of the University of Heidelberg and the Institutional Review Board of the Navrongo Health Research Centre in Ghana.

### Cost estimation

Annual costs for the year 2010 were calculated for each of the twelve health centres. While there are several methods in costing health care services, a systematic review of costing methods conducted by Mogyorosy et al. (2005) concluded that no method is better than the others in all criteria. Rather, the choice of method depends on available data, the study setting and other factors [8]. Taking these criteria into consideration, we used the step-down allocation (SDA) approach as discussed in the WHO manual [9] and Conteh (2004) and used in other studies [10-12].

### Step down allocation approach

The step-down allocation (SDA) approach is a method in which the resources necessary to run a health centre are first identified and then allotted to selected cost centres or service departments on an allocation basis [13,14]. The costs centres are usually ranked according to importance and the costs are being assigned to centres lower down the scale in a step-wise fashion [8]. Using SDA approach, we followed six main steps: (1) defining the final product, (2) defining cost centres, (3) identifying the full cost for each input, (4) assigning inputs to cost centres, (5) allocating all costs to final cost centres, and (6) computing total and unit costs.

Accordingly, costs for each of the twelve health centres were calculated using full costing approach, which required the calculation of both capital and recurrent costs. We defined the final product as antenatal and delivery services.

Secondly, we defined cost centres/categories based on the functions of the departments. Three cost centres were identified: direct, intermediate and indirect costs centres. Direct costs centres, or final cost centres, refer to the cost of services provided directly to patients [10,13,15]. It is the final level of cost centres that Conteh refers to as the ‘end-points of the production line’ [13]. In our study, the direct cost centres were the ANC, delivery services and other care (e.g. inpatient, outpatient, family planning, etc.). Intermediate cost centres refer to costs of diagnostic and departmental support to the direct cost centres, which we identified as the pharmacy and laboratory departments. The indirect cost centres or overhead costs refer to costs incurred by departments or activities that provide services only to other departments of the health care centre, not directly to patients. In this category we included administration and vehicle costs for our analysis.

In the third step, we identified the full cost for each input. We generated a list of all resources and grouped them into the following categories (Table 2)-personnel, administration, pharmacy, vaccines, laboratory, medical supplies (representing recurrent or running costs of health centres), building, vehicle and equipment costs (representing capital costs). The total annual costs were estimated by multiplying and summing quantities consumed or available on each specific item by the unit price. In addition, donated items were valued and included in the cost calculation.

Capital costs, resources whose lifespan is greater than one year, were also calculated. There are several methods of measuring and valuing capital costs in economic evaluation, but the best method is to annuitize capital outlay over the useful life asset, which represents the equivalent annual cost [14]. Capital costs were annualized using a discount rate with their respective useful life years. A discount rate of 3% was chosen in conformity with most economic evaluation studies conducted in developed and developing countries [9,10,16,17] and in the absence of any accepted alternative rate used in Ghana. Based on expert opinion and literature review a useful life of 10 years was used for equipment and 30 years for building [9-11,18].

In the fourth step, we assigned the categorized costs to the three cost centres. Some costs can be assigned immediately to certain cost centres. Accordingly, personnel, administration, pharmacy, laboratory and vehicle costs were assigned directly to the relevant cost centres. Personnel cost was assigned first as it accounted for the largest share of costs. In addition, medical supplies, building cost, equipment were distributed to direct cost centres.

In the fifth step, we reallocated all costs from the two cost centres (indirect and intermediate cost centres) to the direct cost centres (ANC, delivery and others). In the final step, we calculated unit costs. The allocated costs for each direct cost centre were divided by the outputs of each of these centres. Unit cost (average cost) refers to the cost of providing a single good or service [13]. Accordingly, the unit cost for ANC was calculated by dividing the total cost of ANC over the total number of ANC consultations conducted in the year irrespective of the number of women who made multiple visits. The unit cost for delivery was calculated by dividing the total cost of delivery over the total number of deliveries in the year.

### Other calculations

We estimated the number of ANC visits based on the recommended minimum of four visits for focused ANC by dividing the total number of ANC visits by 4. All costs in this study were collected in local currency, Ghana cedis (GH₵), and were converted to US$ during data analysis using the average exchange rate for 2010 (1US$= GH₵1.5).

### Sensitivity analysis

One-way sensitivity analysis was conducted to explore the impact of changes of variables that are susceptible to change over time or in different settings so as to assist in the generalization of the study results. This was done by changing each variable through the range of interest or assumptions, while holding other variables constant. Thus, all variables remained constant except the variable of interest.

Given that several studies use 3-5% discount rates, sensitivity analysis was done to assess the impact of using a higher discount rate (5%). The life span of buildings was also varied (from 30 years to 20 years). In addition, given that countries are embarking on several interventions and reforms to encourage women to attend ANC and deliver at the health centre, we expect a future increase in utilization of ANC and delivery services at the health centres. A sensitivity analysis was therefore performed assuming a 10% increase in ANC visits and delivery. Thereafter the threshold for significance was set at 10% change in costs or higher.

## Results

### Cost distribution

The average cost (capital and recurrent costs) of operating the 12 primary health centres (Table 3) in a year was estimated as $136,014 US (median= $132,178 US), with a range from $27,727 US to $389,985 US. The Wiaga Health Centre had the highest overall health care cost ($389,985 US) with the highest average costs for ANC ($86,286 US) and delivery ($38,002 US) as well. This is not surprising as the health centre is large with more staff, more resources and a larger catchment area compared with the other health centres (Table 1).

**Table 3:** Cost distribution by health care centre (US$)

	**Recurrent cost**					**Capital cost**			
**Health care centre**	**Personnel**	**Administration**	**Pharmacy**	**Laboratory**	**Medical supplies**	**Building**	**Vehicle**	**Equipment**	**Total**
Paga	31125.4	10197.6	70116.2	2133.3	2310.9	302.9	4332.2	5505.9	126024
Chiana	75818.2	15083.1	80853.3	1010	8512.2	143.6	7012.3	7771.1	196204
KNE	37370.7	6732.7	8713.4	379.3	1505.9	410	3791.1	3125.2	62028.3
Sirigu	42368	5436.4	28728.1	3007.3	3902.9	108.6	4668.3	5616.9	93836.4
NHC	60672	4726.7	66708.1	-	875.7	252.9	-	7247.9	140483
Kolgo	25422.7	8525.2	88929.3	4668	2453.8	245.7	4519.8	3567.6	138332
Chuchulga	51720	10398.2	13310.1	-	1479.1	102.1	1079.8	2005.5	80094.8
Kanjaga	56468	5963.9	3524.3	-	1072.8	112.1	1329.9	3501.5	71972.4
Siniensi	113768	18848.8	6800.6	-	1289	98.6	2962.7	4773.7	148541
Doninga	17024	534.3	5320.4	116.7	426.5	117.9	461.7	3725.8	27727.3
Wiaga	123852	24054.3	216272	2233.4	5741	215	9276.8	8341.2	389985
Fumbisi	99048	8970.1	40028.4	-	3151.7	102.1	930.7	4708.2	156939
**Total**	**734657**	**119471**	**629304**	**13548**	**32721.8**	**2211.4**	**40365.1**	**59890.3**	1632169
**Average**	**61221.4**	**9955.9**	**52442**	**1129**	**2726.8**	**184.3**	**3363.8**	**4990.9**	136014
**Median**	**54094**	**8747.6**	**34378.2**	**248**	**1908.4**	**130.7**	**3376.9**	**4741**	**132178**

Of the overall total health centre cost, the highest proportion was spent on personnel (45%), followed by pharmacy (39%). Administration (7.3%) and building related costs (0.14%) contributed less than 10% to total health centre costs. By disaggregating the costs, the average recurrent cost (administration, pharmacy, laboratory and medical supplies costs) was estimated as $127,475 US (median= $122,941 US), representing 93.7% of total costs.

### Cost of antenatal care and delivery

The estimated cost attributed to ANC and delivery differed substantially between health centres. The cost attributable to ANC services for all the health centres ranged from $2,707 US to $86,286 US, with an average of $23,063 US (median: $20,997 US). The costs attributable to delivery services ranged from $2,451 US to $38,002 US, with an average of $11,543 US per health centre (Table 4). Overall, ANC (17%) and delivery (8%) comprised less than a quarter of the total cost of operating the health centres. Based on recurrent expenditures, the average cost attributed to ANC and delivery was $20,498 US and $10,349 US respectively.

**Table 4:** Cost and unit cost for antenatal care (ANC) and delivery (US$)

**Antenatal care (ANC)**				**Delivery**		
**Health centres**	**ANC cost for the year 2010 (US$)**	**Number of ANC visits for the year (2010)**	**Unit cost of ANC (US$)**	**Cost of delivery services for the year 2010 (US$)**	**Total number of deliveries for the year (2010)**	**Unit cost of delivery services for the year 2010 (US$)**
**Paga**	2706.54	2725.00	0.99	11590.00	368.00	31.49
**Chiana**	22983.52	1588.00	14.47	5364.00	286.00	18.76
**KNE**	4754.89	2237.00	2.13	5374.00	232.00	23.16
**Sirigu**	8974.69	1605.00	5.59	10413.00	313.00	33.27
**NHC**	25898.67	4074.00	6.36	18578.00	172.00	108.01
**Kolgo**	24534.52	743.00	33.02	7445.00	128.00	58.17
**Chuchulga**	9929.75	1646.00	6.03	2451.00	193.00	12.70
**Kanjaga**	19179.41	1008.00	19.03	15404.00	159.00	96.88
**Siniensi**	22815.24	731.00	31.21	12160.00	80.00	152.00
**Doninga**	7167.94	270.00	26.55	1792.00	70.00	25.60
**Wiaga**	86285.59	1755.00	49.17	38002.00	259.00	146.73
**Fumbisi**	41526.17	1580.00	26.28	9940.00	191.00	52.04
**Total**	**276756.93**	**19962.00**	**220.83**	**138513.00**	**2451.00**	**758.81**
**Average**	**23063.08**	**1663.50**	**18.40**	**11542.75**	**204.25**	**63.23**
**Median**	**20997.33**	**1596.50**	**16.75**	**10176.50**	**192.00**	**42.66**

### Antenatal visits, deliveries and unit costs

The average number of antenatal care (ANC) visits was 1,664 per health centre. Based on the recommended minimum of four visits for focused ANC, the estimated average number of women seeking regular ANC was 416. The average number of deliveries was 204, indicating that about 49% of women who attend ANC delivered at a health facility.

The number of ANC visits and deliveries varied substantially among health centres. For example, while only 270 ANC visits were recorded at Doninga Health Centre, there were as many as 4,074 visits at Navrongo Health Centre (Table 4). In addition, while only 70 deliveries were registered in Doninga, there were 368 deliveries at Paga Health Centre (Table 4). Based on average ANC visits (1,664) and deliveries (204) for the study period, health care centres spent an average of $18.40 US per ANC visit and $63.20 US per delivery, although these costs varied widely across health care centres. An ANC visit at the Paga Health Centre cost only $0.99 US while it cost $49.17 US at the Wiaga Health Centre (Table 4).

Similarly, while the estimated cost of delivery was $12.70 US at the Chuchulga Health Centre, it was $152 US at the Siniensi Health Centre. This difference could be attributed to the number of deliveries, as the smaller the number of deliveries, the higher per head cost, and vice versa. Considering recurrent expenditure alone, the average cost per ANC visit was $1.30 US and the average cost per delivery was $56.60 US per health centre.

The results from the sensitivity analysis (Table 5) show that varying the discount rate applied to capital costs from 3% to 5% and life expectancy of buildings from 30 years to 20 years has very little impact on costs (1%). However, increasing the number of ANC visits and deliveries (by 10%) has much greater impact on cost. For instance, increasing the number of ANC visits by 10% (from 1,663.5 to 1,830) resulted in a 32% reduction in cost per ANC visit (from $18.4 US to $12.6 US), and increasing deliveries by 10% (from 204.25 to 224.68) reduced the unit cost per delivery by 18.75% (from $63.23 US to 51.37 US). This shows that increasing utilization at health centres helps reduce unit costs leading to optimal utilization of the resources.

**Table 5:** Sensitivity analysis

**Variables**	**Capital cost (US$)**	**Total cost (US$)**	**ANC cost (US$)**	**Delivery cost (US$)**	**Unit cost per ANC (US$)**	**Unit cost per delivery (US$)**
***Discount rate***						
3% discount rate	8538.90	136014	23063.08	11542.75	18.40	63.23
*5% discount rate	9278.87	136754	23167.88	11593.89	18.50	63.52
Percentage change (%)	7.97	0.54	0.45	0.44	0.55	0.45
***Building***						
*Using 20 years life span	8594.44	136, 069.60	23069.93	11546.33	18.41	63.26
Percentage change (%)	0.65	0.04	0.03	0.03	0.07	0.04
***Antenatal care (ANC)***						
Using average number of ANC visits =1663.5					18.4	
*10% increase in number of ANC visits = 1830					12.60	
Percentage change (%)					31.51	
***Delivery***						
Using number of deliveries =204.25						63.23
*10% increase in delivery =224.68						51.37
Percentage change (%)						18.75

*Sensitivity analysis variables.

## Discussion

This study analyzed the costs associated with running a health care centre as well as the specific costs associated with ANC and delivery services. Related studies in the past did not include capital costs in their analysis [12,19] and some studies report results for hospitals only [12]. This study therefore provides empirical evidence to health centre managers and policy makers on the full costs of running a primary health centre and how much is spent on ANC and delivery services. This can help to assess how well resources are used in different health centres and whether these services are sufficiently funded.

The study followed the basic principles and steps of costing health care services recommended by WHO [9] and used in similar studies [10-13]. Though various methods are used in costing health care services, the differences reflect different decision situations and the research objective and not due to disagreement on methodology and concepts [8,15].

The study result showed that there is a significant variation in costs across the selected health centres. This results from differences in patient attendance and available resources at the health centres. The average cost of operating a health centre in our study is higher than the national average spending on health per capita of $75 US in 2011 [20]. Furthermore, in comparison with cost studies in other settings, the annual cost of health centres included in this study is higher. In neighbouring Burkina Faso for example, the average recurrent cost of running a health centre is $63,818 US [10], which is about half of the average recurrent expenditure in this study. However, since the Burkina Faso study was conducted 10 years ago, the cost difference could be due to increment in staff salaries, general price increases resulting from the depreciation of the Ghana cedi relative to the US dollar over the period (62% depreciation between 2000–2010) [21] and higher gross domestic product (GDP) per capita in Ghana ($3,100 US) than Burkina ($1,500 US) [22].

In this study, personnel cost emerged as the most significant cost in running a health centre. This is consistent with other provider cost studies, where personnel cost was above 50% of the total cost [12,15,23].

The costs attributed to ANC and delivery differ remarkably among the health centres, and the results show that the utilization rate for ANC was higher than for delivery at the health centres. Similar findings have been reported in other studies [24,25]. The total ANC and delivery costs represent less than a quarter of the total budget of the health centres, which could be attributed to the underutilization of the services, especially for delivery. Unit cost per ANC visit and normal delivery differ as well among the twelve health centres.

The unit cost per ANC visit using recurrent cost is much lower than observed in other cost studies. For example, while the average recurrent cost per ANC visit in our study was $1.30 US, the average recurrent expenditure per ANC visit was estimated at $2.21 US in Uganda, $3.23 US in Malawi, and $2.97 US in Ghana [19]. However, average recurrent expenditure per delivery in this study was estimated at $57 US–which is high when compared with $2.71 US in Uganda, $10.22 US in Malawi and $7.66 US in Ghana [19]. Unlike in other studies [23], our findings reveal that higher total costs result in high unit costs, which is due to low ANC and delivery utilization. As the number of ANC visits or deliveries increases, the economies of scale dictate that the unit costs will tend to be less.

The National Health Insurance Scheme (NHIS) of Ghana enables its members to access care in any accredited public, private or mission-run health facility without paying out-of-pocket for services within its benefit package. The scheme then reimburses the providers at a predetermined rate per service provided. The NHIS reimburses the health centres at a rate of $3.50 US per ANC service rendered to its clients and $8 US per normal delivery [26]. Compared to estimates from this study, the average amounts spent by the health centres per patient receiving ANC and delivery services are higher than the NHIS reimbursement rates for these services. Only two health centres (Paga and KNE) have costs per ANC visit that are within the NHIS antenatal/postnatal reimbursement rate. As for the unit cost per normal delivery, none of the 12 health centres’ estimated unit cost fell within the NHIS reimbursement rate. This has implications for cost recovery by the health care centres [27].

### Limitations of the study

The sample size of twelve health centres in this study represents the results only from these centres and may not be generalizable to other regions in the country. Likewise, record keeping and health information systems at the health centres were poor and could affect the study findings. In some instances, we had to rely on estimations, which could lead to either over-estimation or underestimation.

Also, because the health centres were heterogeneous and might differ in efficiency, the total average cost used in the analysis might not be a reliable estimate of actual output cost. In addition, the cost data was based on expenditure of the health care centres and might not reflect the actual cost of producing the outputs such as ANC and delivery.

As few studies have published data on the costs of MNC, it is difficult therefore to meaningfully compare pre-existing data to our study results. This difficulty is also due to the period of data collection, differences in costing methods, differences in prices and resources across settings and general differences in the economic environment. Despite these challenges, the methodology used in this study is applicable across various settings, and the results are relatively reliable and may serve as a starting point for future cost studies.

## Conclusions

This study demonstrated that with painstaking effort, health facility costing is possible in rural settings and the methodology used is appropriate and can be adopted in conducting similar study in another area. The high unit costs reflect underutilization of the existing capacities of the health centres and, therefore, the need to encourage patients to use the health centres. High utilization of ANC and delivery services should improve efficient use of scarce resources and cost recovery for health centres. The study provides useful information that could be used for cost effectiveness analyses of maternal and neonatal care interventions, as well as for policy makers to make appropriate decisions regarding the allocation and sustainability of health care resources.

### QUALMAT

The QUALMAT research project funded as part of the 7th Framework Programme of the European Union (grant agreement 22982) is a collaboration between the Centre de Recherche en Santé de Nouna (Burkina Faso), Ghent University (Belgium), Heidelberg University (Germany), Karolinska Institute (Sweden), Muhimbili University of Health and Allied Sciences (Tanzania), and Navrongo Health Research Centre (Ghana). The overall objective of this research is to improve the motivation and performance of health workers and ultimately the quality of pre-natal and maternal care services. The intervention packages include the development and implementation of a system of performance based incentives and a computer-assisted clinical decision support system based on WHO guidelines. The interventions are evaluated in a pre-post controlled study design in rural Burkina Faso, Ghana and Tanzania, between 2009 and 2014.

### Consent

Written informed consent was obtained from respondents for the publication of this report and any accompanying images.

## Competing interests

The authors declare not to have any financial and non-financial competing interests.

## Authors’ contributions

MAD contributed to the concept and design of the study, the acquisition of data, data analysis, interpretation of the data, and drafting of the manuscript. PA, GS, HS, HD and SL assisted in the design of the study, development of the data collection instrument and contributed to the write up of the manuscript. JW assisted in the design of the study. RS assisted in the concept and design of the study. All authors read and approved the final manuscript.

## Pre-publication history

The pre-publication history for this paper can be accessed here:

http://www.biomedcentral.com/1472-6963/13/287/prepub
